# Impact of Combined Subthalamic Nucleus and Substantia Nigra Stimulation on Neuropsychiatric Symptoms in Parkinson's Disease Patients

**DOI:** 10.1155/2017/7306192

**Published:** 2017-01-26

**Authors:** U. Hidding, A. Gulberti, A. Horn, C. Buhmann, W. Hamel, J. A. Koeppen, M. Westphal, A. K. Engel, C. Gerloff, D. Weiss, C. K. E. Moll, M. Pötter-Nerger

**Affiliations:** ^1^Department of Neurology, University Medical Center Hamburg-Eppendorf, 20246 Hamburg, Germany; ^2^Department of Neurophysiology and Pathophysiology, University Medical Center Hamburg-Eppendorf, 20246 Hamburg, Germany; ^3^Department of Neurosurgery, University Medical Center Hamburg-Eppendorf, 20246 Hamburg, Germany; ^4^Department for Neurodegenerative Diseases and Hertie Institute for Clinical Brain Research, University of Tübingen, 72076 Tübingen, Germany

## Abstract

The goal of the study was to compare the tolerability and the effects of conventional subthalamic nucleus (STN) and combined subthalamic nucleus and substantia nigra (STN+SNr) high-frequency stimulation in regard to neuropsychiatric symptoms in Parkinson's disease patients. In this single center, randomized, double-blind, cross-over clinical trial, twelve patients with advanced Parkinson's disease (1 female; age: 61.3 ± 7.3 years; disease duration: 12.3 ± 5.4 years; Hoehn and Yahr stage: 2.2 ± 0.39) were included. Apathy, fatigue, depression, and impulse control disorder were assessed using a comprehensive set of standardized rating scales and questionnaires such as the Lille Apathy Rating Scale (LARS), Modified Fatigue Impact Scale (MFIS), Becks Depression Inventory (BDI-I), Questionnaire for Impulsive-Compulsive Disorders in Parkinson's Disease Rating Scale (QUIP-RS), and Parkinson's Disease Questionnaire (PDQ-39). Three patients that were initially assigned to the STN+SNr stimulation mode withdrew from the study within the first week due to discomfort. Statistical comparison of data retrieved from patients who completed the study revealed no significant differences between both stimulation conditions in terms of mean scores of scales measuring apathy, fatigue, depression, impulse control disorder, and quality of life. Individual cases showed an improvement of apathy under combined STN+SNr stimulation. In general, combined STN+SNr stimulation seems to be safe in terms of neuropsychiatric side effects, although careful patient selection and monitoring in the short-term period after changing stimulation settings are recommended.

## 1. Introduction

Neuropsychiatric symptoms in Parkinson's disease (PD) represent a common, disabling, occasionally socially disruptive condition; they are difficult to treat and have a major impact on quality of life [1, 2]. Emotional neuropsychiatric symptoms represent a spectrum of various phenomena including apathy, fatigue, and depression on the one hand and impulse control disorder and mania on the other hand [1, 3]. Emotional symptoms are related to several factors such as environmental and cultural influences or personality traits. Of particular importance is the relation of neuropsychiatric symptoms to the disease itself, as in the case of apathy and depression, and the relation to the antiparkinsonian treatment observed for impulse control disorder [1, 4]. Apathy, fatigue, and depression are common and disabling symptoms preceding the onset of motor symptoms by several years [5]. In the pathophysiological understanding of neuropsychiatric symptoms, observations of early alpha synuclein pathology of nondopaminergic brainstem nuclei such as the locus coeruleus (LC) and the raphe nuclei have shed new light on the role of brainstem nuclei and basal ganglia-brainstem projections [6, 7]. Particularly, the noradrenergic LC moved into the focus of interest, since the LC is interconnected via widespread ascending projections to cortical, subcortical, cerebellar, brainstem, and spinal cord circuits [8]. According to this broad efferent linking, the LC is involved in numerous functions, such as arousal, regulation of the sleep-wake-cycle, attention, behavioral flexibility, memory, posture, gait and balance, and emotions [9, 10]. The LC might therefore represent an important structure in the pathogenesis of certain neuropsychiatric symptoms such as apathy, fatigue, or depression.

Some neuropsychiatric symptoms might represent a contraindication for deep brain stimulation (DBS) in the subthalamic nucleus (STN) such as severe depression. Despite contradictory observations [11–13], some symptoms such as impulse control disorder have the chance to ameliorate with STN-DBS in well selected patients [14, 15].

Preoperatively, it has to be differentiated whether the neuropsychiatric symptom is mainly due to the disease itself or due to the dopaminergic medication [4]. After STN-DBS surgery, the reduction of dopaminergic medication, including dopamine agonists, might worsen certain hypodopaminergic neuropsychiatric symptoms such as apathy or depression or improve hyperdopaminergic symptoms such as impulse control disorders. Still, the effect of DBS on neuropsychiatric symptoms is varied and complex in the literature indicating a challenging interaction of DBS and emotional symptoms [4, 16].

**Figure 1:**
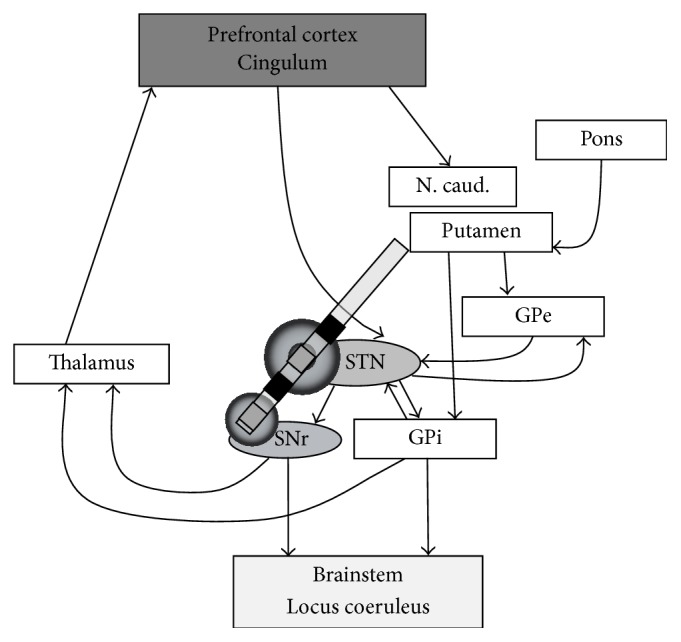
Simplified model of DBS induced modulation of the limbic basal ganglia loops including brainstem projections. N. caud.: nucleus caudatus; GPe: globus pallidus externus; GPi: globus pallidus internus; STN: nucleus subthalamicus; SNr: substantia nigra pars reticulata.

Recently, increased interest has been focused on the basal ganglia-brainstem projections in the mediation of STN-DBS beneficial effects [17, 18]. In the current basal ganglia model of PD, it is assumed that the pathologically “overactive” STN induces an increased inhibition of subcortical basal ganglia routes to brainstem centers via increased activation of the inhibitory globus pallidus internus (GPi) and substantia nigra pars reticulata (SNr) [17, 19]. It has been proposed that STN-DBS normalizes the efferent basal ganglia output and results in disinhibition of brainstem activity (see Figure 1) [20, 21]. In view of apparently untreatable, residual, mainly brainstem-mediated symptoms such as axial symptoms and gait disorders under STN-DBS, efforts have been made to costimulate the substantia nigra [22]. The assumption of an additional benefit of SNr stimulation is based on the hypothesis that there might be still residual overactivity of SNr in PD with STN-DBS resulting in ongoing increased GABAergic inhibition of brainstem nuclei which would be supplementarily suppressed by simultaneous high-frequency stimulation within the SNr. The substantia nigra pars reticulata is of particular interest since animal data suggest dense reciprocal GABAergic interconnections between the SNr and mesencephalic brainstem centers such as the pedunculopontine nucleus [23, 24] and consecutively the LC in the downstream. In a recent double-blinded cross-over study, costimulation of the STN and SNr induced an improvement of freezing of gait without effects on depression (assessed by BDI) and impulsiveness (assessed by Barrett Impulsiveness Scale) [22]. Other neuropsychiatric symptoms such as apathy and fatigue were not yet systematically investigated.

The aim of the current study was to compare the costimulation of STN and SNr to conventional STN stimulation in terms of certain LC associated neuropsychiatric symptoms such as apathy, fatigue, and depression. In particular, we intended to demonstrate the safety of a combined STN+SNr stimulation, namely, that STN+SNr stimulation would not worsen neuropsychiatric symptoms in PD patients treated with DBS.

## 2. Materials and Methods

All statistical values in this section are given as mean ± standard deviation (SD).

### 2.1. Participants

Twelve patients (1 female; age: 61.3 ± 7.3 years) suffering from advanced idiopathic PD (disease duration: 12.3 ± 5.4 years; Hoehn and Yahr stage: 2.2 ± 0.39 in the MED-ON and STN-DBS-ON condition [25]) participated in the study (see Table 1). The inclusion criteria for this study were the following: (1) PD patients with bilateral electrode implantation in the STN for DBS for at least 3 months; (2) the deepest contacts of the implanted electrodes having to be positioned within the dorsal aspect of the SNr (see below for stereotactic reconstruction technique); (3) unchanged dopaminergic medication in the preceding 4 weeks; (4) MoCA score at baseline ≥ 20 [26]; (5) good effect of conventional STN stimulation on motor symptoms (response to STN stimulation > 30%). There was no further stratification of PD patients, for example, in terms of severity of freezing of gait or neuropsychiatric parameters except dementia.

Preoperatively, all PD patients were screened and selected for DBS surgery in accordance with common guidelines [27]. Patients showed a significant improvement of the motor subscore (III) of the Movement Disorder Society Unified Parkinson's Disease Rating Scale (MDS-UPDRS) after intake of soluble levodopa (MED-OFF 39.0 ± 17.3, MED-ON 10.5 ± 6.3, improvement of 72%). Preoperatively, the levodopa equivalent daily dose (LEDD) was 921.0 ± 348.5 mg (conversion factors used for the calculation of LEDD according to Tomlinson et al. 2010 [28]). Neuropsychiatric or medical comorbidities as well as MRI abnormalities were preoperatively excluded. Postoperatively, at the time of baseline measurement, the motor subscore (III) of MDS-UPDRS in “Dopa-on” with STN-DBS-OFF was 34.4 ± 6.7. The postoperative LEDD was 707.5 ± 240.3 mg. Further clinical details are summarized in Table 1. All participants had to read and sign an information consent before participation into the study. The study was approved by the local ethics committee and was conducted in agreement with the Code of Ethics of the World Medical Association (Declaration of Helsinki, 1967).

### 2.2. Electrode Implantation and Contact-Position Reconstruction

Surgical details and a precise description of the microelectrode (MER) guided mapping procedure of the subthalamic region are given elsewhere [29, 30]. Briefly, the dorsal STN was targeted 11-12 mm lateral to midline, 0–3 mm posterior to the midcommissural point, and 1–3 mm inferior to the intercommissural plane. Preoperative high-resolution T2-weighted magnetic resonance images (fused with stereotactic computed tomography (CT) scans) were used to adjust target coordinates in individual patients. The majority of patients (*n* = 9) were awake during stereotactic DBS electrode placement. In three cases, DBS surgery was carried out under general anesthesia with propofol and remifentanil [31]. In all cases, final electrode placement was guided by the results of intraoperative MER and test stimulation. To this end, up to five parallel tracks (3 ± 1) were used to map the subthalamic region with sharp tungsten electrodes (AlphaOmega, Nazareth, Israel; impedance, 595 ± 167 kOhm). Unit activity was amplified and high-pass-filtered between 300 Hz and 6 kHz and stored for offline analysis (NeurOmega, AlphaOmega, Nazareth, Israel). The sensorimotor STN was identified by cell responses to passive and active movements and a high prevalence of oscillating unit activities in the range between 10 and 30 Hz. Differentiation of STN from SNr was based on established electrophysiological criteria. Specifically, the decrease of background noise and the replacement of irregular STN unit activity by tonic regular high-frequency spiking of SNr neurons marked the ventral exit of the STN and dorsal aspect of the SNr, respectively. For all tracks that traversed the target nuclei, we first reconstructed the 3D location of the ventral STN border and the dorsal SNr relative to the midcommissural point. Then we determined the Euclidean distance of these positions to the ventral DBS electrode contact. The localization of the implanted electrode contacts (model 3389, Medtronic, Minneapolis, Minnesota, USA, in 8 cases, and 8-poled electrode model, Boston Scientific, Valencia, CA, USA, in 2 cases) was determined by coregistration of the preoperative T1 magnetic resonance imaging (MRI) scans and postoperative CT scans using commercially available software (iPlan stereotaxy; Brainlab, Feldkirchen, Germany). A detailed description of the reconstruction of single electrode contacts is provided elsewhere [32, 33]. According to stereotactic atlases, high-resolution MRI, and MER-guided mapping, the upper border of the SNr is positioned 4.5–6 mm below the plane in between anterior and posterior commissure [22].

### 2.3. Design

**Figure 2:**
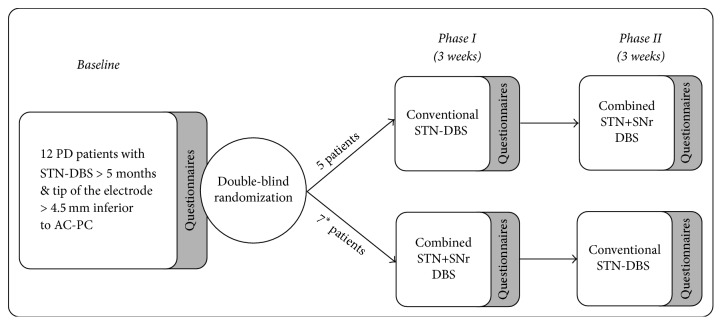
Study design: randomized cross-over trial over 6 weeks. After baseline assessment, PD patients were assigned in phase I to either conventional STN-DBS or combined STN+SNr stimulation. After 3 weeks, patients were switched to the other stimulation mode. (*∗*) Three of the 7 patients starting the phase I with a combined STN+SNr-DBS withdrew within the first week.

The study design was a single center, randomized, double-blind, cross-over clinical trial to compare the effects of STN stimulation versus combined STN+SNr stimulation as described previously [22]. Blinded patients were investigated at three visits with a time interval of three weeks in between (baseline recording, phase I and phase II, see Figure 2). All visits were performed in a medication-on condition and included motor tests and questionnaires concerning different aspects of everyday life during the preceding 3 weeks. Motor tests and questionnaires were performed by blinded investigators. After testing thresholds for side effects in the SNr, defined stimulation settings for the STN and STN+SNr stimulation were fixed for the course of the experiment. The STN settings were not different from those before the study. Afterwards, stimulation of the STN or combined STN+SNr was set by a nonblinded investigator in a randomized manner for the following three weeks; that is, 5 of 12 PD patients received first a conventional STN stimulation (the control stimulation for placebo effects) and after three weeks the combined STN+SNr stimulation, while the other 7 patients received first the combined STN+SNr stimulation followed by conventional STN stimulation (see Figure 2). After completion of phase I, the second visit was performed with reprogramming of stimulation parameters in a cross-over manner for the following three weeks (starting phase II). The third visit was performed after six weeks when phase II was completed. Patients were unblinded and the preferred stimulation mode was programmed as permanent therapeutic stimulation. Medication and stimulation parameters were held constant during phase I and phase II of the study. Only in one case did the stimulation amplitude in the SNr have to be reduced after two days due to dyskinesias.

### 2.4. Questionnaires and Outcome Measures

The goal of the study was to investigate the tolerability and the effect of a combined STN+SNr stimulation with regard to neuropsychiatric issues like apathy, fatigue, depression, and impulse control disorders (ICDs). For this aim, we used the Lille Apathy Rating Scale (LARS [34]), Modified Fatigue Impact Scale (MFIS [35, 36]), Becks Depression Inventory (BDI-I [37]), and the Questionnaire for Impulsive-Compulsive Disorders in Parkinson's Disease Rating Scale (QUIP-RS [38, 39]). To evaluate a possible impact of different stimulation settings on the quality of life of the patients, the Parkinson's Disease Questionnaire (PDQ-39) was applied [40].

The LARS is designed to diagnose and measure severity of apathy symptoms. The score is based on a structured interview including 33 items divided into 9 domains (everyday productivity, interests, taking the initiative, novelty seeking, motivation, voluntary actions, emotional responses, concern, social life, and self-awareness, each domain ranging from −4 to 4 points). The global score ranges from −36 to +36, with higher scores indicating a higher degree of apathy. Cut-off values range from −36 to −22 for nonapathetic, from −21 to −17 for slightly apathetic, and from −16 to −10 and from −9 to +36 for moderate and severely apathetic patients, respectively [34]. To the best of our knowledge, at the moment there is no study assessing the LARS sensitivity to changes in apathy over a shorter interval of time than 3 months [34, 41–43].

The MFIS consists of 21 items and provides an assessment of the global effects of fatigue expressed as a total score and, more specifically, in terms of physical, cognitive, and psychosocial functioning as subscores. In particular, all items are scaled so that higher scores indicate a greater impact of fatigue on personal activities. The total MFIS score ranges from 0 to 84, while the physical subscale can range from 0 to 36, the cognitive subscale from 0 to 40, and psychosocial subscale from 0 to 8. Schiehser et al. (2013) tested 100 nondemented PD patients at mild-moderate stages with the MFIS, collecting a mean total score of 32 points [44]. To the best of our knowledge, a specific cut-off score for the MFIS in PD patients could not be established yet [44].

The BDI-I is a 21-item questionnaire to assess depressive symptoms with scoring of 0–3 for each item. A maximum of 63 points can be achieved (higher score indicating worse symptoms). A score of 16 is considered the cut-off for mild depression in PD [45].

The QUIP-RS is a standardized instrument for detecting and assessing impulse control disorders. It has four main questions (concerning frequently reported thoughts, urges/desires, and behaviors related to ICDs), each applied to four ICDs (compulsive gambling, buying, eating, and sexual behavior) and 3 related disorders (medication use, punding, and hobbyism). It displays a five-point Likert scale (score of 0–4 for each question) to assess the frequency of behaviors. The total QUIP-RS score can range from a minimum of 0 to a maximum of 112 points. The cut-off points for individual ICDs (possible score of 0–16 for each ICD) are as follows: gambling ≥ 6; buying ≥ 8; sex ≥ 8; and eating ≥ 7. For combined ICDs (possible score of 0–64), a QUIP-ICD score can be calculated and the cut-off point is ≥10. Hobbyism-punding (possible score of 0–32) has a cut-off point of ≥7 [39].

The Parkinson's Disease Questionnaire (PDQ-39) is a 39-item self-report questionnaire, which records frequent and specific health-related problems over the last month and reproduces reliably the quality of life of Parkinson patients [40]. Besides, the Parkinson's disease summary index (PDSI) provides an index of the global impact of PD on health status of the patients. The PDQ-39 assesses how often PD patients experience difficulties across 8 different dimensions of quality of life: mobility, activities of daily living, emotional well-being, stigma, social support, cognition, communication, and bodily discomfort. The PDSI is derived by the sum of the eight PDQ-39 scale scores divided by eight (the number of subscales), which yields a score between 0 and 100 (higher scores indicating impaired quality of life). This is equivalent to expressing the sum of all 39 item responses as a percentage score [46].

### 2.5. Statistics

Since three patients withdrew from the study due to intolerance of combined STN+SNr-DBS, ANOVAs were calculated for the 9 patients that completed the study. This number of patients is in line with analog DBS studies with similar intends, limitations, and approaches [14, 22, 47–49]. The scores as collected at the three experimental visits were compared: (1) baseline, (2) standard STN-DBS, and (3) combined STN+SNr-DBS. Repeated measures ANOVAs with the within-subjects factor DBS (baseline, STN-DBS, and STN+SNr-DBS) were performed separately for each test (BDI-I, LARS, QUIP-RS, QUIP-ICD, MFIS, and PDQ39) with PASW Statistics (PASW Statistics for Mac, version 18.0, SPSS Inc., Chicago, IL, USA). For interaction effects, Greenhouse–Geisser corrected *p* values were calculated if sphericity was violated (Mauchly's sphericity test). Alpha level was set at 0.05. Post hoc comparisons were performed with Wilcoxon signed rank test (Matlab 7.10 and associated toolboxes, MathWorks, Natick, MA). For scales with validated cut-off levels for PD (i.e., BDI-I, LARS, and QUIP-ICD), Fisher's exact tests were performed on a 2 × 2 contingency table with categories “kind of DBS-stimulation” × “symptomaticity.”

## 3. Results

Descriptive statistics in the main text include mean ± standard deviation (SD) of the mean, while in the boxplots median, interquartile range (IQR), whiskers (highest/lowest values of the dataset within 1.5 times the IQR), and outliers (<1st percentile and the >99th percentile) are illustrated.

### 3.1. Stereotactic Reconstruction of Recording Positions and Electrode Contacts

For this study, MER data was successfully retrieved from 23/24 hemispheres (due to technical reasons, MER could not be performed on the left hemisphere of one patient). 59/71 tracks (83%) traversed the STN. SNr unit activity was recorded on 52/71 tracks (73%). In most cases, either the central (15/24, 63%) or the anterior trajectory (7/24, 29%) was chosen for permanent implantation of DBS electrodes. The remaining two electrodes were implanted in the posterior and lateral trajectory, respectively.

**Figure 3:**
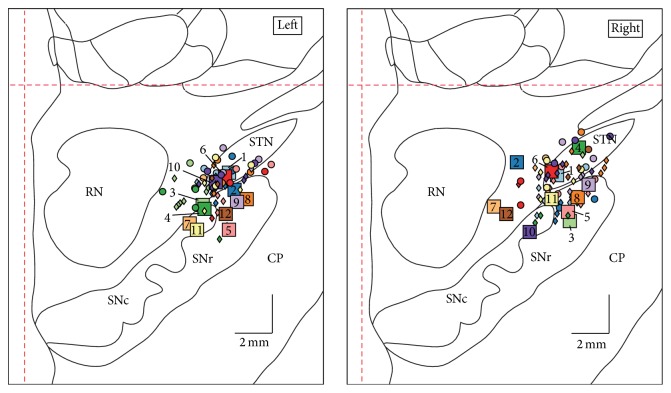
Stereotactic reconstruction: squares indicate the localization of the most ventral DBS electrode contacts, superimposed on frontal sections of the stereotactic atlas of Morel, at a level 5 mm behind midcommissural point [50]. Circles indicate the ventral STN border as defined by standard electrophysiological criteria. Diamonds indicate the presence of unambiguous SNr cell activity on MER tracks. Colors represent individual patients. The dashed red lines denote midline and AC-PC level, respectively. STN: subthalamic nucleus; SNr: substantia nigra pars reticulata; SNc: substantia nigra pars compacta; CP: cerebral peduncle; RN, red nucleus.

Figure 3 shows the localization of the reconstructed most ventral DBS contacts (squares), which were used for combined STN+SNr stimulation, in relation to the MER-defined ventral STN border (circles) and nigral recording sites (diamonds) for every patient.

The ventral DBS electrode contact was located significantly closer to the dorsal SNr (1.9 ± 1 mm) compared to the ventral border of the STN (2.4 ± 1.4 mm; paired *t*-test, *p* = 0.02). The stereotactic coordinates of the most ventral DBS contact relative to the midcommissural point (MCP; mean ± SD in mm) were *x* = 10.4 ± 0.9, *y* = 2.8 ± 1.3, and *z* = 6.3 ± 1.0 for the left hemisphere and *x* = 10.1 ± 1.7, *y* = 2.7 ± 1.5, and *z* = 5.7 ± 1.4 for the right side (*x*: lateral to midline; *y*: posterior to MCP; *z*: inferior to AC-PC level). Furthermore, in the majority of the cases studied here (16/23, 70%), the depth (*z*-axis) of the ventral DBS electrode contact was located below the MER-defined dorsal level of the substantia nigra. This quite deep position of the electrode occurred as part of the normal variation during routine care.

### 3.2. Stimulation Effects on Apathy

**Figure 4:**
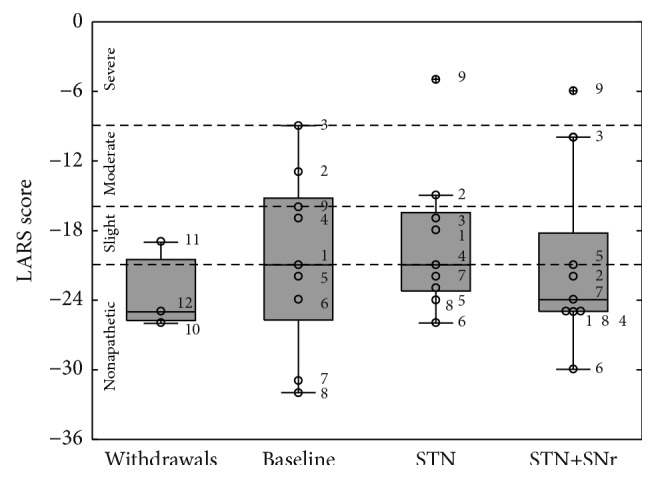
Boxplots of LARS scores: the box plot depicted on the left represents the baseline values of the three patients who withdrew. The other three box plots represent the values of the 9 PD patients who have completed the study at the three experimental time points: (1) baseline, (2) STN alone, and (3) combined STN+SNr-DBS. The numbered circles represent the single patients as listed in Table 1. The dashed horizontal lines represent the cut-offs as previously described.

PD patients of the investigated cohort were slightly apathetic at baseline as measured by the LARS (−20.56 ± 7.73 for the nine patients completing the study; −23.33 ± 3.79 for the three withdrawals). The mean degree of apathy of PD patients was not significantly modulated by factor DBS [*F*(2,16) = 0.491; *p* = 0.621]. The scores during conventional STN-DBS (−19.00 ± 6.32) and combined STN+SNr-DBS (−20.89 ± 7.78) remained in a similar range (see Figure 4). Post hoc comparisons did not reveal any significant significance between the stimulation conditions.

According to the cut-off criteria for the LARS, at baseline, six patients of twelve patients were nonapathetic, three patients showed a slight apathy, two patients were moderately apathetic, and one was severely apathetic. During conventional STN-DBS in the study, four of nine patients were nonapathetic, while three patients showed a slight apathy, one a moderate apathy, and one a severe apathy. With the combined STN+SNr-DBS-stimulation, six of nine patients were nonapathetic, one patient was slight apathetic, one patient was moderately apathetic, and one patient was severely apathetic. Fisher's exact tests performed on a 2 × 2 contingency table with categories “kind of DBS-stimulation” × “apathy” did not show any significant change in terms of apathy between the different stimulation modes (*p* = 1 for baseline versus STN-DBS alone; *p* = 0.637 for baseline versus combined STN+SNr-DBS; *p* = 0.637 for STN-DBS alone versus combined STN+SNr-DBS, Fisher's exact tests). Thus, there was no significant change of overall mean apathy scores considering the total cohort, although single cases might have had benefit. A nonparametric Spearman's correlation performed with the MoCA scores and the LARS scores at baseline did not reveal a possible interplay between apathy and the cognitive state of the patients (rho = −0.297; *p* = 0.437).

### 3.3. Stimulation Effects on Fatigue

Fatigue represents sometimes the only symptom that apathetic patients complain about [1]; these two symptoms seem to be closely associated.

**Figure 5:**
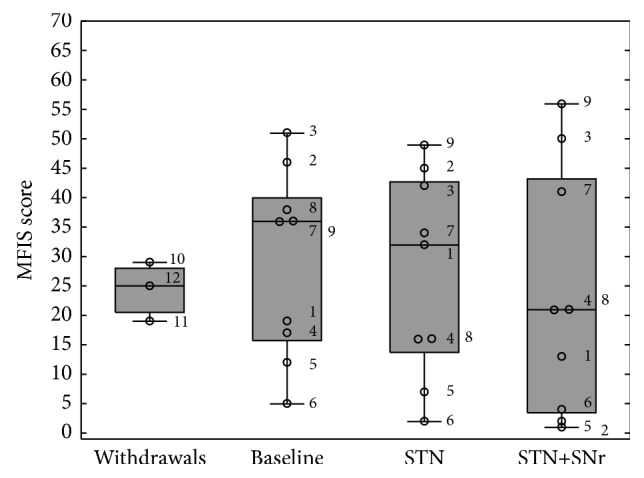
Boxplots of MFIS scores: the box plot depicted on the left represents the baseline values of the three patients who withdrew. The other three box plots represent the values of the 9 PD patients who have completed the study at the three experimental time points: (1) baseline, (2) STN alone, and (3) combined STN+SNr-DBS. The numbered circles represent the single patients as listed in Table 1.

For the nine patients completing the study, the mean MFIS score at baseline was 28.89 ± 16.05 (24.33 ± 5.03 for the three withdrawals). The general level of fatigue was not significantly influenced by DBS [*F*(2,16) = 0.607; *p* = 0.557] (Figure 5). Compared to conventional STN-DBS (27.00 ± 17.23) there was a slight reduction of fatigue with combined STN+SNr-DBS (23.22 ± 21.02), which was not significant in post hoc analyses. Further ANOVAs performed separately for the three MFIS subscales (i.e., physical, cognitive, and psychosocial fatigue) did also not reveal any significant modulation of fatigue subscores due to different DBS modes (for all ANOVAs performed on the above-mentioned 3 subscales [*F*(2,16) ≤ 1.020; *p* ≥ 0.383]).

### 3.4. Stimulation Effects on Depression

**Figure 6:**
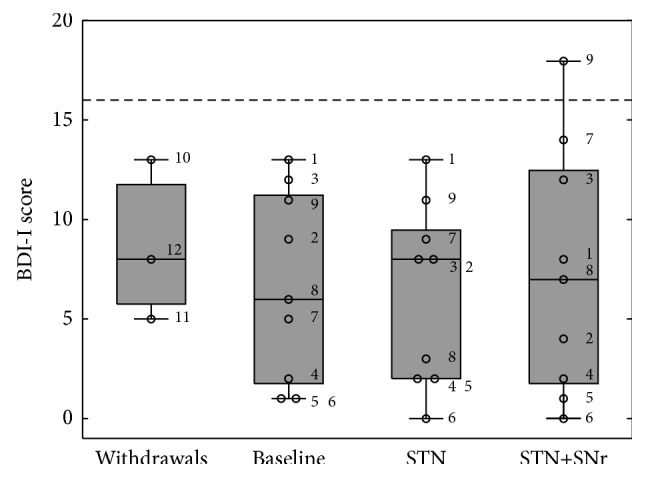
Boxplots of BDI-I scores: the box plot depicted on the left represents the baseline values of the three patients who withdrew. The other three box plots represent the values of the 9 PD patients who have completed the study at the three experimental time points: (1) baseline, (2) STN alone, and (3) combined STN+SNr-DBS. The numbered circles represent the single patients as listed in Table 1. The dashed horizontal line represents the cut-off for PD patients as previously discussed in Material and Methods.

The recruited PD patients did not reveal depressive symptoms at baseline (three withdrawals: 8.67 ± 4.04; nine patients completing the study: 6.67 ± 4.77). The BDI-I score was not modulated by factor DBS [*F*(2,16) = 0.378; *p* = 0.691] meaning that neither STN-DBS (6.22 ± 4.57), nor STN+SNr stimulation (7.33 ± 6.26) worsened depressive symptoms (Figure 6). Post hoc comparisons did not reveal any significant difference between the two stimulation conditions.

Fisher's exact tests performed on a 2 × 2 contingency table with categories “kind of DBS-stimulation” × “depression” did not show any significant change in terms of symptoms at the three experimental time points (*p* = 1 for all three comparisons).

### 3.5. Stimulation Effect on Impulse Control Disorders

**Figure 7:**
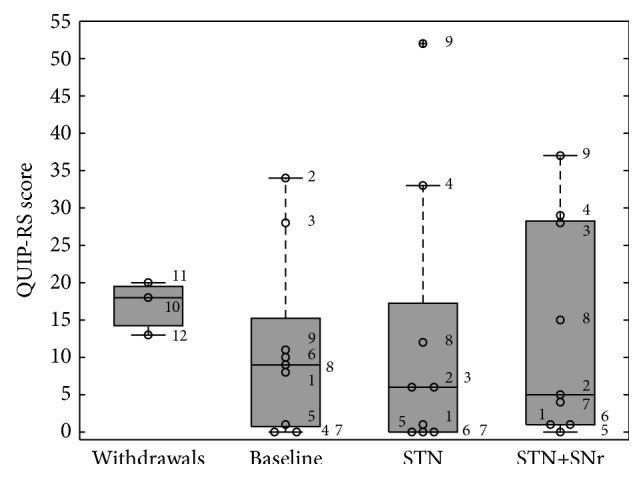
Boxplots of QUIP-RS scores: the box plot depicted on the left represents the baseline values of the three patients who withdrew. The other three box plots represent the values of the 9 PD patients who have completed the study at the three visits: (1) baseline, (2) STN alone, and (3) combined STN+SNr-DBS. The numbered circles represent the single patients as listed in Table 1. A cut-off for the total QUIP-RS score is not available.

**Figure 8:**
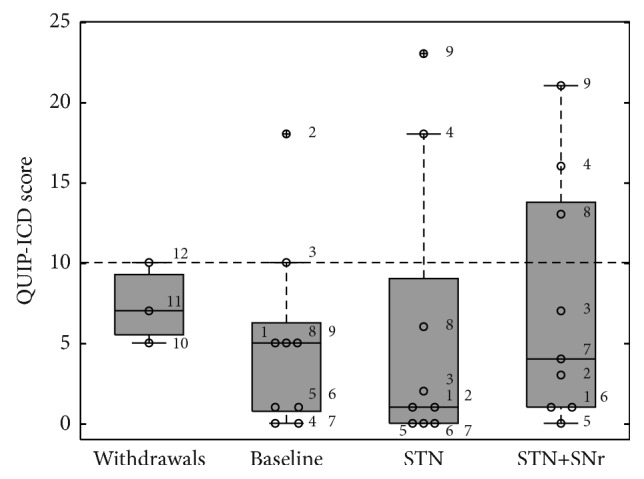
Boxplots of combined QUIP-ICD scores: the box plot depicted on the left represents the baseline values of the three patients who withdrew. The other three box plots represent the values of the 9 PD patients who have completed the study at the three experimental time points: (1) baseline, (2) STN alone, and (3) combined STN+SNr-DBS. The numbered circles represent the single patients as listed in Table 1. For the combined QUIP-ICD score the cut-off point is ≥10.

To measure the impact of the different DBS conditions on impulse control disorders, both the QUIP-RS total score (Figure 7) and the combined QUIP-ICD score (Figure 8) have been analysed.

The QUIP-RS total score at baseline was 11.22 ± 12.11 (for the three withdrawals 17.00 ± 3.61), while at control STN stimulation was 12.22 ± 18.25. The QUIP-RS total score with the combined STN+SNr-DBS was 13.33 ± 14.41. ANOVA testing of QUIP scores did not reveal any modulation due to the DBS mode [*F*(2,16) = 0.065; *p* = 0.850]. Planned post hoc comparisons did also not reveal any significant contrast between stimulation conditions.

Regarding the combined QUIP-ICD score (Figure 8), PD patients did also not reveal any modulation of scores for impulse control disorders due to the DBS mode, as measured at the three experimental visits [*F*(2,16) = 0.331; *p* = 0.602]. The QUIP-ICD score at baseline was 5.00 ± 5.87 (7.3 ± 2.52 for the three withdrawals), while at control STN stimulation was 5.67 ± 8.7. The QUIP-ICD score with the combined STN+SNr-DBS was 7.33 ± 7.57. Fisher's exact tests performed on a 2 × 2 contingency table with categories “kind of DBS-stimulation” × “ICD” did not show any significant change in terms of ICD behaviors between the different stimulation modes (*p* = 1 for all three comparisons). Also for the QUIP-ICD score, as for the QUIP-RS total score, planned post hoc comparisons did not reveal any significant score modulation due to different stimulation regimes.

### 3.6. Stimulation Effects on Quality of Life

**Figure 9:**
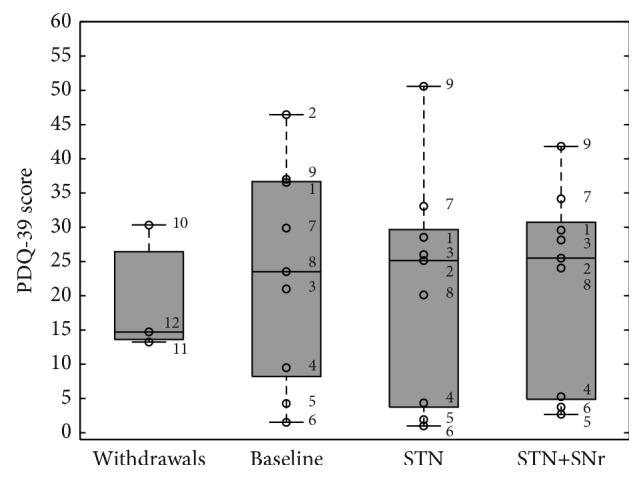
Boxplots of PDQ-39 scores expressed as Parkinson's disease summary index (PDSI) scores: the box plot depicted on the left represents the baseline values of the three patients who withdrew. The other three box plots represent the values of the 9 PD patients who have completed the study at the three experimental time points: (1) baseline, (2) STN alone, and (3) combined STN+SNr-DBS. The numbered circles represent the single patients as listed in Table 1.

As measured by means of PDQ-39 (Figure 9), quality of life was not significantly influenced by DBS modes in the PD cohort [*F*(2,16) = 0.374; *p* = 0.596] with scores of 23.33 ± 15.70 (19.46 ± 9.46 for the three withdrawals) at baseline, 21.21 ± 16.42 with STN stimulation, and 21.68 ± 14.30 with STN+SNr-DBS. Planned comparisons by means of the Wilcoxon signed rank test showed nonsignificant differences for the PDSI scores at the different visits. Further ANOVAs separately performed on the different 8 PDQ-39 subscales for STN-DBS versus the combined STN+SNr-DBS did not reveal significant differences (for all ANOVAs performed for the 8 subscores [*F*(2,16) ≤ 2.519; *p* ≥ 0.112]).

## 4. Discussion

In this study, combined STN+SNr stimulation was noninferior compared to STN stimulation in terms of neuropsychiatric symptoms assessed in this study in PD patients. There were no significant differences between these two stimulation conditions in terms of mean scores of apathy, fatigue, depression, impulse control disorder, and quality of life, although individual cases profited from the combined stimulation mode in terms of apathy. In PD patients with low neuropsychiatric morbidity in terms of apathy, fatigue, depression, and impulsivity at baseline, the combined STN+SNr stimulation mode did not induce or deteriorate emotional symptoms in accordance with previous reports [22]. Currently, STN+SNr stimulation is predominantly applied in PD patients with specific motor symptoms to improve freezing of gait [22, 51]. The results confirm safety and good tolerability of combined STN+SNr stimulation applied in otherwise neuropsychologically healthy, carefully selected PD patients who are treated with this stimulation mode for improvement of freezing of gait, for instance.

Three patients withdrew from the study prematurely due to side effects. All three patients terminated the study under a combined STN+SNr-DBS regime, suggesting that for these patients this stimulation mode was not adequate or even disadvantageous. In detail, side effects were worsening of motor functions as well as a lack of beneficial effects of levodopa (case  10), akathisia (case  11), and increased confusion and hallucinations (case  12). From baseline neuropsychiatric measurements and electrode position, these three patients did not differ from the other PD patients who completed the study. In the interpretation of the results, one needs to consider the limits of the study with a small number of patients, gender disparity, and the lack of comparison to controls. Another constraint may be represented by a nonsensitivity of the LARS to changes in apathy over a short period of 3 weeks [34, 41–43]. Further investigations with a larger, controlled patient cohort need to evaluate whether certain neuropsychiatric characteristics of PD patients may be red flags to select or not select patients to undergo combined STN+SNr stimulation.

There are some rare and selected reports of stimulation induced neuropsychiatric side effects of ventral limbic STN stimulation [52] or SNr stimulation [53, 54] such as stimulation induced mania or even depression. In a recent trial, combined interleaving STN+SNr stimulation with application of different, adapted amplitudes in both nuclei was safe in terms of neuropsychiatric side effects [22]. This finding could be reproduced in our cohort of PD patients which completed the study. The stimulation associated neuropsychiatric effects are divergent and are proposed to be dependent on the preexisting medical and psychiatric condition [53], the exact electrode position within the nuclei, and the voltage used for stimulation. High-amplitude stimulation might be associated with some overlapping effects on the limbic part of the STN and the dorsal SNr due to the extended electrical field, so adjustment of amplitudes, for example, in the interleaving stimulation mode seems to be favorable for the combined stimulation.

Recent work has focused on the description and analyses of postoperative apathy, fatigue, and depression [1–3]. Apathy is one of the most common and disabling neuropsychiatric symptoms in PD with a prevalence of 15–70% depending on disease severity [55, 56]. It can be defined as a lack of motivation, interests, and emotions resulting in decreased goal-directed behaviors. Several studies in early PD have shown that apathy correlates with more severe motor impairment, suggesting an underlying common mechanism such as hypodopaminergic drive [56]. It has been proposed that, in the spectrum of dopamine-dependent symptoms, apathy is a neuropsychiatric correlate of the motor symptom akinesia, impulse control disorder, a nonmotor counterpart of dyskinesia [3].

Certain neuropsychiatric symptoms are differentially associated with specific domains of cognitive decline in PD [57–59]. The cohort of enrolled PD patients was predominantly cognitively normal in screening tests at baseline and had low scores in regard of apathy, depression, fatigue, and impulsivity. In this cohort of patients, we did not find specific, significant effects of combined STN+SNr stimulation.

Postoperatively after STN-DBS, the reduction of the dopaminergic medication differentially influenced hypo- and hyperdopaminergic symptoms [3]. While akinesia is sufficiently improved with STN-DBS with low L-Dopa dosage [60–63], apathy and depression are not. Dyskinesias and impulse control disorders might improve both with postoperative LEDD reduction. Apathy might be difficult to treat, since there is insufficient response to antidepressants [39] and increase of postoperative LEDD for apathy treatment might tilt the “dopaminergic scale” and reinforce dyskinesia and impulse control disorder.

Dopamine seems to be a key player in the pathogenesis of Parkinsonian apathy, but it is probably not the only neurotransmitter involved. In a recent study, beside dopaminergic denervation, serotonergic involvement within the basal ganglia network was detected in apathetic de novo PD patients [64]. Noradrenergic involvement might be also involved in emotional and cognitive domains in Parkinson's disease [65]. Such observations justify the attempt to facilitate nondopaminergic brainstem nuclei such as the LC to improve neuropsychiatric symptoms in PD.

In some individual cases, apathy was slightly improved by the combined STN+SNr stimulation mode, but there was no comparable trend for depression, fatigue, or impulse control disorder. This divergent effect might be due to different underlying anatomical loops mediating diverse emotional manifestations [1]. PET and structural and functional MRT studies revealed in depressed PD patients anatomical changes of the temporal lobe, orbitofrontal cortex, and anterior cingulate cortex [66] associated with reduced connectivity in cortico-subcortical loops reflecting a disturbed top-down regulation of cortico-limbic emotional loops [67, 68]. In apathetic PD patients, there are anatomical changes of several cortical areas such as the precentral gyrus, inferior parietal gyrus, inferior frontal gyrus, insula, cingulum, and precuneus [69] as well as the nucleus accumbens [70]. Functional connectivity reductions were detected in left-sided circuits, predominantly involving limbic striatal and frontal territories [71]. Further brain structures might be involved in the complex nature of apathy with bottom up- and downregulation as derived from observations in health and disease [3, 72]. Those limbic circuits were demonstrated to be influenced by STN-DBS as shown for, for example, the right frontal gyri in PET studies [73]. Thus, STN+SNr stimulation might differentially modulate different cortico-subcortical loops.

Brainstem and probably the LC might be involved in the pathophysiology of apathy, fatigue, and depression [8, 74, 75]. It is suggested that SNr stimulation might increase the drive of basal ganglia-brainstem projections by the release of pathological inhibition of brainstem centers such as LC activity (Figure 1). This model focuses on direct basal ganglia output structures such as the brainstem which are predominantly modulated by DBS. The constraint of this model is the neglect of large-scale remote circuits which might be also involved in the pathogenesis of apathy or depression. Further evaluation of DBS effects on those remote circuits needs to be assessed in PD patients with neuropsychiatric complications.

It remains to be investigated in a larger cohort of apathetic, depressed PD patients with fatigue whether costimulation of STN and SNr might be superior to STN stimulation in impacting LC associated neuropsychiatric symptoms such as apathy, fatigue, and depression by additional “pushing” basal ganglia-brainstem projections without the need to change the postoperative LEDD. If so, the combined STN+SNr stimulation mode represented a routinely applicable therapeutic option to overcome postoperative hypodopaminergic neuropsychiatric complications to improve quality of life.

## Figures and Tables

**Table 1:** Clinical and demographic characteristics of PD patients.

Case, gender, age	Disease duration [years]	Time with DBS [months]	LEDD [mg]	MoCA score at baseline	DBS System	STN-DBS parameters	Combined STN+SNr-DBS parameters	*X*, *Y*, *Z* coordinates
						Left electrode Right electrode	Left electrode Right electrode	Left electrode Right electrode
1, M, 61	23	54	1150	30	ME	Left: 1− 2− G+, 3.5 V, 60 *μ*s, 125 Hz Right: 9− 10− G+, 2.7 V, 60 *μ*s, 125 Hz	Left: 1− 2− G+, 3.5 V, 60 *μ*s, 125 Hz; 0− G+, 2.0 V, 60 *μ*s, 125 Hz Right: 9− 10− G+, 2.7 V, 60 *μ*s, 125 Hz; 8− G+, 2.0 V, 60 *μ*s, 125 Hz	Left: 10.9, 2.2, 4.7 Right: 10.5, 3.8, 4.7

2, M, 63	23	105	860	26	ME	Left: 1− G+, 1.9 V, 60 *μ*s, 125 Hz; 2− G+, 2.9 V, 60 *μ*s, 125 Hz Right: 9− G+, 1.9 V, 60 *μ*s, 125 Hz; 10− G+, 3.3 V, 60 *μ*s, 125 Hz	Left: 2− G+, 2.9 V, 60 *μ*s, 125 Hz; 1− 0− G+, 1.9 V (1.5 V), 60 *μ*s, 125 Hz Right: 10− G+, 3.3 V, 60 *μ*s, 125 Hz; 8− 9− G+, 1.9 V (1.5 V) 60 *μ*s, 125 Hz	Left: 11.2, 1.9, 5.6 Right: 8.3, 5.5, 4

3, M, 56	9	36	880	26	ME	Left: 1+ 2− G+, 2.2 V, 60 *μ*s, 125 Hz Right: 10− G+, 4.3 V, 60 *μ*s, 125 Hz	Left: 2− G+, 2.2 V, 60 *μ*s, 125 Hz; 0− G+, 1.0 V, 60 *μ*s, 125 Hz Right: 10− G+, 4.3 V, 60 *μ*s, 125 Hz, 8− G+, 1.0 V, 60 *μ*s, 125 Hz	Left: 9.5, 2.8, 6.4 Right: 11.2, 1.4, 7.2

4, M, 67	16	60	600	23	ME	Left: 1− G+, 1.5 V, 60 *μ*, 125 Hz Right: 9− 10− G+, 3.9 V, 60 *μ*s, 125 Hz	Left: 1− G+, 1.5 V, 60 *μ*, 125 Hz; 0− G+, 2.0 V, 60 *μ*s, 125 Hz Right: 9− 10− G+, 3.9 V, 60 *μ*s, 125 Hz; 8− G+, 2.0 V, 60 *μ*s, 125 Hz	Left: 9.6, 4.7, 6.6 Right: 11.7, 3.1, 3.2

5, M, 65	9	9	300	28	ME	Left: 1− G+, 2.8 V, 60 *μ*s, 125 Hz Right: 9− G+, 3.0 V, 60 *μ*s, 125 Hz	Left: 1− G+, 2.8 V, 60 *μ*s, 125 Hz; 0− G+, 1.5 V, 60 *μ*s, 125 Hz Right: 9− G+, 3.0 V, 60 *μ*s, 125 Hz; 8− G+, 1.5 V, 60 *μ*s, 125 Hz	Left: 10.9, 1.4, 7.7 Right: 11.1, 2.7, 6.7

6, M, 74	9	9	360	22	ME	Left: 1− G+, 2.7 V, 130 Hz Right: 9− G+, 2.6 V, 60 *μ*s, 130 Hz	Left: 1− G+, 2.7 V, 60 *μ*s, 125 Hz; 0− G+, 1.5 V, 60 *μ*s, 125 Hz Right: 9− G+, 2.9 V, 60 *μ*s, 125 Hz; 8− G+, 1.5 V, 60 *μ*s, 125 Hz	Left: 10.7, 2.6, 4.9 Right: 10.2, 2.5, 4.5

7, M, 51	9	15	900	27	BS	Left: 2− 30%, 3− 70%, 3.4 mA, 60 *μ*s, 125 Hz Right: 10− 20%, 11− 80%, 4.0 mA, 60 *μ*s, 125 Hz	Left: 1− 23%, 2− 23%, 3− 54%, 4.4 mA, 60 *μ*s, 125 Hz Right: 9− 20%, 10− 16%, 11− 64%, 5.0 mA, 60 *μ*s, 125 Hz	Left: 8.81, 3.38, 7.37 Right: 7.04, 4.28, 6.41

8, M, 57	7	18	580	27	BS	Left: 3− 70%, 4− 30%, 4.5 mA, 60 *μ*s, 130 Hz Right: 12− 100%, 3.8 mA, 60 *μ*s, 130 Hz	Left: 3− 61%, 4− 26%, 1− 13%, 5.2 mA, 60 *μ*s, 130 Hz Right: 12− 85%, 9− 15%, 4.5 mA, 60 *μ*s, 130 Hz	Left: 11.85, 3.37, 6.09 Right: 11.63, 2.69, 5.9

9, M, 71	11	13	600	27	ME	Left: 1− G+, 3.5 V, 60 *μ*s, 125 Hz Right: 9− G+, 2.7 V, 60 *μ*s, 125 Hz	Left: 1− G+, 3.5 V, 60 *μ*s, 125 Hz; 0− G+, 1.0 V, 60 *μ*s, 125 Hz Right: 9− G+, 2.7 V, 60 *μ*s, 125 Hz; 8− G+, 1.0 V, 60 *μ*s, 125 Hz	Left: 11.34, 2.18, 6.24 Right: 12.23, 0.2, 5.22

10, F, 66	9	5	700	25	ME	Withdrawal, experimental phase not performed	Left: 2− G+, 2.4 V, 60 *μ*s, 125 Hz; 0− G+, 0.7 V, 60 *μ*s, 125 Hz Right: 11− G+, 2.5 V, 60 *μ*s, 125 Hz; 8− G+, 0.7 V, 60 *μ*s, 125 Hz	Left: 10.2, 0.9, 5.2 Right: 9, 0.6, 7.8

11, M, 55	13	23	700	28	ME	Withdrawal, experimental phase not performed	Left: 3− G+, 2.9 V, 60 *μ*s, 125 Hz; 0− G+, 0.7 V, 60 *μ*s, 125 Hz Right: 10− G+, 2.9 V, 60 *μ*s, 125 Hz; 8− G+, 0.7 V, 60 *μ*s, 125 Hz	Left: 9.2, 2.8, 7.7 Right: 10.2, 2.4, 6

12, M, 53	10	16	860	24	BS	Withdrawal, experimental phase not performed	Left: 3− G+, 2,7 mA, 60 *μ*s, 119 Hz; 1− G+, 0.7 mA, 60 *μ*s, 119 Hz Right: 12−/13− G+, 4,7 mA, 60 *μ*s, 119 Hz; 9− G+, 0.7 mA, 60 *μ*s, 119 Hz	Left: 10.73, 5.34, 6.87 Right: 7.72, 3.14, 6.82

“Disease duration [years]” is calculated from the date of the first diagnosis to the date of baseline measurement of the experiment. “DBS parameters” include active contacts, amplitude (volts or mA), pulse width (microseconds), and stimulation frequency (Hz), for the left and right electrode, respectively. Electrode coordinates are given as mm lateral to the midline (*X*), posterior to the midcommissural point (*Y*), and inferior to the intercommissural plane (*Z*). Note that the deepest contacts were contacts 0 and 8 (Medtronic) or contacts 1 and 9 (Boston Scientific). LEDD: levodopa equivalent daily dose; ME: Medtronic; BS: Boston Scientific.
